# Sensitive, quantitative detection of *Besnoitia darlingi* and related parasites in intermediate hosts and to assess felids as definitive hosts for known and as-yet undescribed related parasite species

**DOI:** 10.1016/j.ijppaw.2020.01.011

**Published:** 2020-01-24

**Authors:** Gereon Schares, Jitender P. Dubey, Benjamin Rosenthal, Mareen Tuschy, Andrea Bärwald, Franz J. Conraths

**Affiliations:** a Friedrich-Loeffler-Institut, Federal Research Institute for Animal Health, Institute of Epidemiology, Südufer 10, 17493, Greifswald, Insel Riems, Germany; b Animal Parasitic Diseases Laboratory, Beltsville Agricultural Research Center, Agriculture Research Service, United States Department of Agriculture, Beltsville, USA

**Keywords:** *Besnoitia darlingi*, *Besnoitia neotomofelis*, *Besnoitia oryctofelisi*, Primer, Besnoitiosis, Rodent, Lagomorph, Real-time PCR

## Abstract

*Besnoitia darlingi, B. neotomofelis* and *B. oryctofelisi* are closely related coccidian parasites with cats as definitive hosts. While *B. darlingi* uses opossums as intermediate hosts, *B. neotomofelis* and *B. oryctofelisi* have been described in Southern Plains woodrats (*Neotoma micropus*) from the USA and in domestic rabbits from Argentina, respectively. A comparison of the Internal Transcribed Spacer-1 (ITS-1) region of the ribosomal DNA (rDNA) of these *Besnoitia* spp. showed only a few differences. The present study aimed at developing a real-time PCR to detect *B. darlingi, B. neotomofelis* and *B. oryctofelisi* in tissues of intermediate and in faeces of definitive hosts in order to support studies of these organisms’ epidemiology and pathogenesis.

The established PCR was based on primer regions distinct from the ITS-1 sequences of ungulate *Besnoitia* spp. and made use of a Besnoitia universal probe. To monitor inhibition, a heterologous internal control was established based on the enhanced green fluorescent protein gene. The real-time PCR reacted with *B. darlingi*, *B. neotomofelis* and *B. oryctofelisi,* while the novel PCR did not recognize ungulate *Besnoitia* spp. (*B. besnoiti, B. bennetti, B. tarandi*). DNA of Apicomplexa ascribed to other Besnoitia-related genera, including other gut parasites of cats (*Cryptosporidium parvum*, *Giardia duodenalis*, *Tritrichomonas foetus*), was not recognized. The real-time PCR had an analytic sensitivity of less than 1 tachyzoite per reaction. In feline faeces spiked with *B. darlingi* oocysts, the limit of detection was a DNA amount equivalent to 1 oocyst per PCR reaction. In *B. darlingi* infected ɣ-interferon knock-out mice, the lung was identified as the predilection organ. In conclusion, this real-time PCR should advance further studies on these parasites and may inspire research on related species, not only in the Americas, but also in other parts of the world.

## Highlights

• A quantitative real-time PCR was established to detect *Besnoitia darlingi*.
• Analytical sensitivity of the real-time PCR for tachyzoites and oocysts was high.
• Lung tissue was the predilection site for *B. darlingi* in ɣ-interferon knockout mice.
• Related parasites like *B. neotomofelis* and *B. oryctofelisi* are detected by this PCR, too.
• PCR holds promise to assess the epidemiology of *B. darlingi* and related species.

## Graphical abstract

**Figure:**
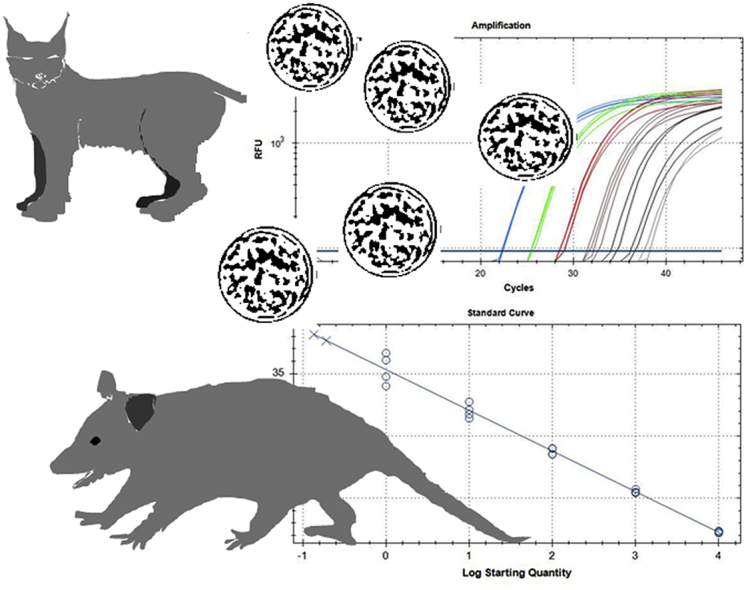

## 1 Introduction

*Besnoitia darlingi, B. neotomofelis* and *B. oryctofelisi* are closely related coccidian parasites, for which cats have been established as definitive hosts (Dubey et al., 2003a; Dubey and Yabsley, 2010; Smith and Frenkel, 1977, 1984). Whereas *B.* *darlingi* uses opossums (*Didelphis virginiana*) as intermediate hosts, *B. neotomofelis* and *B. oryctofelisi* have been described in the Southern Plains woodrat (*Neotoma micropus*) in the USA and in domestic rabbits from Argentina, respectively (Dubey and Yabsley, 2010; Smith and Frenkel, 1984; Venturini et al., 2002).

Another closely related Besnoitia species, *B. jellisoni*, was initially described in the USA, with the white-footed deer mouse (*Peromyscus maniculatus*) and three species of kangaroo rats (*Dipodomys* species) as intermediate hosts (Ernst et al., 1968; Frenkel, 1953). In contrast to *B. darlingi, B. neotomofelis* and *B. oryctofelisi*, the definitive host of *B. jellisoni* is unknown. Domestic cats, other carnivorous mammals, but also various birds and snakes have been excluded as final hosts (Frenkel, 1977; Wallace and Frenkel, 1975).

A comparison of the Internal Transcribed Spacer-1 (ITS-1) region of the ribosomal DNA (rDNA) of all these *Besnoitia* spp. of New World marsupials, rodents and domestic rabbits showed only a few differences (Olias et al., 2011; Verma et al., 2017). Nevertheless, the ITS-1 ribosomal gene locus of Besnoitia species shows informative nucleotide variances. Phylogenetic analysis clearly separated those *Besnoitia* spp. detected in small rodents, marsupials and rabbits from those *Besnoitia* spp. of ungulates (*B. besnoiti*, *B. caprae*, *B. bennetti*, *B. tarandi*) with a genetic divergence in that locus of >20% (Olias et al., 2011).

Thus, the ITS-1 rDNA sequence represented an ideal target to establish PCRs for the detection of genome sequences from such *Besnoitia* spp. of New World marsupials, rodents and domestic rabbits. However, due to the genetic difference relative to the ITS-1 region of *Besnoitia* spp. of ungulates, this region does not appear suitable for establishing a pan-Besnoitia-PCR. Nevertheless, the ITS-1 region represents an interesting target. In analogy to other coccidian parasites such as *Toxoplasma gondii*, it seems likely that the ITS-1 sequence is present more than 100-times in the genome of a single organism (Guay et al., 1992), which may facilitate the development of tests with high analytic sensitivity. The ITS-1 region-based Besnoitia PCRs (endpoint and real-time PCRs) may be used to study the life cycle and biological traits of these parasites and to identify further Besnoitia species of the same clade. Therefore, the present study sought to develop a real-time PCR for the detection of *B. darlingi* and closely-related species like *B. neotomofelis* and *B. oryctofelisi.*

## 2 Material and methods

### 2.1 Parasites

*Besnoitia darlingi* oocysts shed by a naturally-infected bobcat (*Lynx rufus*) (bobcat #20; Verma et al. (2017)) were send to the Friedrich-Loeffler-Institut, Greifswald-Insel Riems, Germany. The bobcat had been obtained through legal trapping by licensed trappers from Mississippi in February 2017 as previously described (Verma et al., 2016).

Tachyzoites of *B. besnoiti* (Bb1Evora03), *N. caninum* (NC-1) and *T. gondii* (RH) were cultivated in MARC-145 cells, isolated and purified as reported previously (Schares et al., 2013). *B. tarandi* (Bt-CA-Quebec 1, Caribou, Canada, Schares et al. (2019)), *B. bennetti* (Texas, USA; S. DaNotta, G. Schares, unpublished), *B. darlingi* (Michigan, USA, Dubey et al. (2002)), *B. neotomofelis* (Texas, USA, Dubey and Yabsley (2010)), *B. oryctofelisi* (Argentina (Dubey et al., 2003a)) were isolated from cell-cultures, as well. Bradyzoites of *Sarcocystis cruzi* (Germany) were obtained from infected tissues from naturally infected cattle (Schares et al., unpublished). Oocysts of *Hammondia heydorni*, *H. hammondi*, *Cystoisospora* spp., *Eimeria bovis* (all from Germany) were obtained by sucrose flotation from the faeces of dogs, cats and cattle as reported previously (Schares et al., 2005). Oocysts of *Cryptosporidium parvum* (Germany) were kindly provided by Prof. Dr. A. Daugschies, Institute of Parasitology Leipzig, Germany). *Tritrichomonas foetus* DNA was supplied by the National Reference Laboratory for tritrichomonosis (Friedrich-Loeffler-Institut, Jena, Germany), *Giardia duodenalis* (Germany) DNA purified from in-vitro cultured trophozoites was kindly provided by Dr. C. Klotz, Robert Koch Institut, Berlin, Germany.

### 2.2 Infection in mice

Mouse experiments (bioassays) reported in this publication were approved by the Landesamt für Landwirtschaft, Lebensmittelsicherheit und Fischerei of the German Federal State of Mecklenburg-Vorpommern (permission 7221.3-2-023/17). A dose of about 300 sporulated oocysts collected from a bobcat naturally infected with *B. darlingi* (bobcat #20; Verma et al. (2017)) was used to inoculate two ɣ-interferon-gene knockout (GKO) mice (C.129S7 (B6)-Ifngtm1Ts/J, The Jackson Laboratory, Bar Harbor, Maine, USA) at the Friedrich-Loeffler-Institut, Greifswald-Insel Riems, Germany. When the mice became ill after 8 or 10 days (i.e. weight loss, ruffled hair), they were humanely euthanized and necropsied under sterile conditions. Half of the heart and lung tissue was homogenized in 1 ml cell-culture medium using a mortar and pestle and 0.5 ml of the homogenate of 1 mouse (i.e. the mouse which developed disease first) inoculated intraperitoneally into another GKO mouse. A cell-culture isolate established from the first mouse that developed disease was designated Bdar-Bobcat#20-FLI.

### 2.3 Cell culture

Homogenized tissues (0.5 ml) were used to initiate a *B. darlingi* infected cell-culture in African green monkey (*Cercopithecus aethiops*) kidney cells (CV-1 cells, ATCC CCL-70; medium DMEM, 2% FCS at 37 °C/5% CO2). The homogenate was added to a confluent cell culture and removed after 4 h. After the cell-culture had been established, it was propagated further by splitting the cultures or by weekly passages of spontaneously-liberated parasites onto new CV-1 cells. Tachyzoites spontaneously released in cell-culture were isolated from medium via filtration through 5 μm filter-units (Minisart single use syringe filter, non-sterile, hydrophilic, Sartorius, Göttingen, Germany) and washed extensively by five rounds of centrifugation (800 *g*, 4 °C, 10 min, no brake) and re-suspension in 14 ml ice-cold phosphate buffered saline (PBS, pH 7.2). The final parasite pellet was stored at −20 °C until use for DNA extraction.

### 2.4 DNA extraction

Oocysts of *Hammondia* spp. and *Cystoisospora* spp. were isolated from faeces using a combined sedimentation and flotation procedure employing 13 ml concentrated sucrose (specific gravity 1.3) to 1 ml faecal sediment as described previously (Schares et al., 2005). Floating oocysts were collected with a wide pipette by adding 1 ml PBS to the top of the sucrose solution, stirring the PBS to bring the oocysts into the PBS phase, followed by carefully collecting up to 2 ml of the solution from the top of the sucrose phase. The oocyst suspension was washed three times by centrifugation (1100 *g*, 7 min, without brake) and a 5- to 10-fold volume of PBS. The DNA of the semi-pure oocysts suspensions (about 10^4^–10^5^ oocysts of *Hammondia* spp., *Cystoisospora* spp., *C. parvum*) was extracted using the NucleoSpin Soil kit (Macherey and Nagel, Düren, Germany).

DNA was extracted from *in-vitro* isolated parasites using commercial kits according to the manufacturers’ instructions (*B. besnoiti*, *B. tarandi*, *B. darlingi*, *B. bennetti, N. caninum*, *T. gondii*, *S. cruzi*: NucleoSpin Tissue, Macherey and Nagel, Düren, Germany; *B. oryctofelisi, B. neotomofelis*: DNeasy Tissue Kit, QIAGEN, Hilden, Germany).

Aliquots of a parasitologically negative feline fecal sample (tested by standard flotation) were spiked with different numbers of sporulated *B. darlingi* oocysts collected from bobcat #20 (i.e. 100, 10, 1 or 0 oocysts per 150 mg faeces). DNA extraction from 150 mg aliquots of faeces was performed using the Quick-DNA Fecal/Soil Microbe DNA Miniprep Kit (Zymo Research Europe GmbH, Freiburg, Germany) according to the manufacturer's recommendations. Extraction resulted in 100 μl DNA per faecal sample.

Mouse tissues (25 mg samples) were extracted using the NucleoSpin Tissue Kit (Macherey and Nagel, Düren, Germany) following the manufacturer's recommendations. The DNA concentration of infected mouse tissue was determined using a Nanophotometer (Implem) and adjusted to 15 ng/μl with water.

All extractions were done along with negative extraction controls to monitor for potential contaminations. The digestion buffer of the respective kits served as a negative processing control.

### 2.5 Endpoint PCR

To test DNA extracted from oocyst samples for the DNA of coccidia, a PCR was performed using the common apicomplexan small subunit ribosomal DNA (SSU-rDNA) primers COC-1 and COC-2 (Ho et al., 1996). Primers were used at a final concentration of 0.5 mM and dNTPs at a final concentration of 250 mM each (Stratec Molecular GmbH, Berlin, Germany). Taq polymerase (Stratec Molecular GmbH, Berlin, Germany) was used at a final concentration of 1U/25 μl with the buffer system supplied with the enzyme. The PCR cycling conditions were 94 °C for 5 min, followed by 10 cycles of 56 °C (with 0.5 °C decrement per cycle after the 1st cycle) for 1 min, 72 °C for 1 min and 94 °C for 1 min, followed by 40 cycles of 51 °C for 1 min, 72 °C for 1 min and 94 °C for 1 min. The PCR ended with an incubation at 51 °C for 1 min and a final extension at 72 °C for 5 min.

To test for Giardia DNA, we used published primers, AS1 GiardiaF, AS2 GiradiaR (Ghosh et al., 2000) and to test for *T.* *foetus* DNA, we used the primers TFR1, TFR2 (Felleisen, 1997). Reagents, except for primers, were the same as described above. They were used in the same concentrations as described for the coccidian PCR. The PCR cycling conditions were 94 °C for 5 min, followed by 35 cycles of 60 °C for 1 min, 72 °C for 1 min and 94 °C for 1 min. The PCR ended with a final extension at 72 °C for 10 min.

### 2.6 Real-time PCR

Quantitative real-time PCR detecting the ITS-1 region was performed as described previously (Schares et al., 2011) with some modifications, including integration of an Internal Control (IC) system (Hoffmann et al., 2006). The novel real-time PCR was subsequently named BdanjoRT1 PCR.

To monitor inhibition in the real-time PCR, a heterologous plasmid DNA, DNA resembling the enhanced green fluorescent protein (EGFP) gene (Hoffmann et al., 2006) was added to the reaction mix including the primers EGFP1-F, EGFP1-R and the probe EGFP1 (Table 1). A 712 bp fragment of the EGFP gene was amplified and cloned into the pGEM-Teasy standard cloning vector (Promega, Walldorf, Germany) in reverse orientation to obtain the IC-2 DNA (pGEM-EGFP2-rev). The amount of the IC-2 DNA added to each reaction was adjusted so that it resulted in a Cq value of about 32 in the real-time PCR.

**Table 1:** Primers, probes and their final concentrations in a novel real-time PCR assay (named BdanjoRT1), established to amplify DNA of *Besnoitia darlingi*, *B. jellisoni*, *B. akodoni*, *B. neotomofelis* and *B. oryctofelisi*.

Assay	Names of primers and probes	Sequences of primers and probes 5′–3′	Modifications of probes	Final concentration	Reference
BdanjoRT1					
	BdanjoFor	CAA CCA TTC AAC CTT TGA ACC C		500 nM	This study
	BdanjoRev	CAC CAT ACT TCC CGA ATG CAC		500 nM	This study
	Bb11-12	CCC TCG AAA CGA GAG ATG CAA GC	5′-FAM, 3′-BHQ1	100 nM	Schares et al. (2011)
Internal control PCR, IC2 PCR					
	EGFP1-F	GAC CAC TAC CAG CAG AAC AC		500 nM	Hoffmann et al. (2006)
	EGFP2-R	GAA CTC CAG CAG GAC CAT G		500 nM	Hoffmann et al. (2006)
	EGFP1	AGC ACC CAG TCC GCC CTG AGC A	5′-HEX, 3′-BHQ1	160 nM	Hoffmann et al. (2006)

Reactions were performed in a final volume of 20 μl using a commercial master-mix (PerfeCTa MultiPlex qPCR ToughMix, Quantabio, VWR International, Darmstadt, Germany) and a CFX96 instrument (Biorad Laboratories GmbH, Munich, Germany). Primers and probes (Table 1) were purchased from MWG-Biotech (Ebersberg, Germany). Standard concentrations for primers (500 nM) and probes (100 nM, Bb11-12; 160 nM, EGFP1) were applied. Concentrations are displayed in Table 1. The cycling conditions in the BdanjoRT1 real-time PCR were 95.0 °C (5 min, initial denaturation), followed by 45 cycles, during which the samples were first incubated at 95.0 °C for 10 s and then at 60.0 °C for 30 s. After each cycle the light emission by the fluorophore was measured. Real-time PCR results were analysed using the CFX manager software Version 1.6 (Biorad Laboratories GmbH, Munich, Germany).

**Fig. 1:**
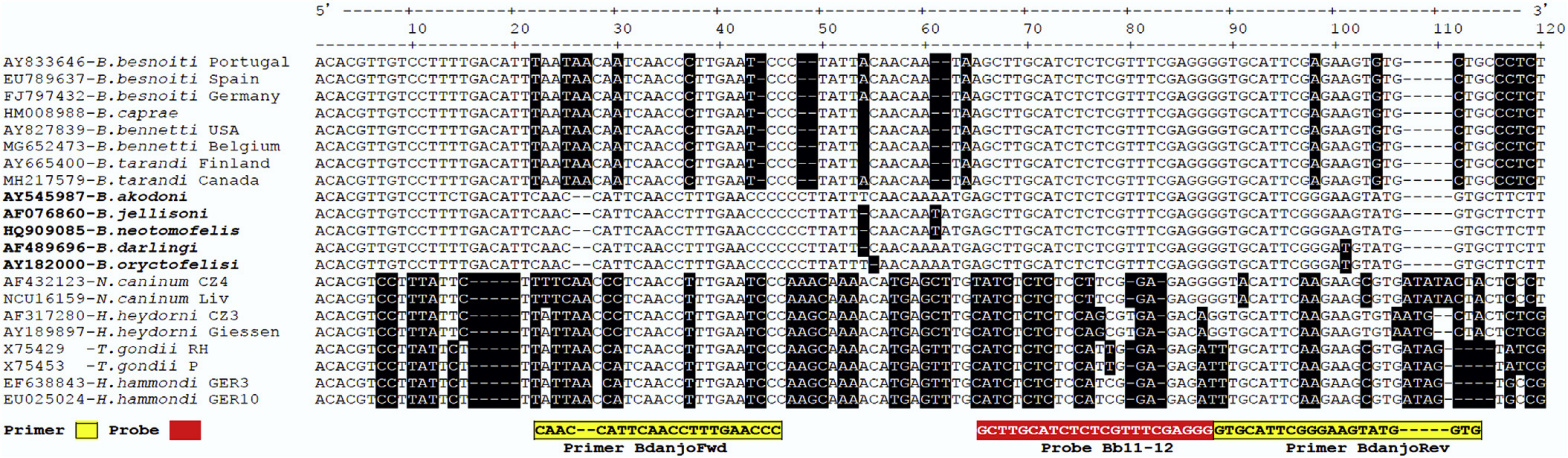
Location of the primers and the probe of the *Besnoitia darlingi*/*B.neotomofelis* /*B. oryctofelisi*-specific real-time PCR assay BdanjoRT1 within the ITS-1 region of the rRNA gene. The sequences of the ITS-1 region of *B. akodoni* (AY545987, bold), *B. jellisoni* (AF076860, bold), *B. neotomofelis* (HQ909085, bold), *B. darlingi* (AF489696, bold) and *B. oryctofelisi* (AY182000, bold), were aligned relative to sequences of other *Besnoitia* spp. including *B. besnoiti* from Portugal, Spain and Germany, *B. bennetti* reported from the USA and Belgium, *B. tarandi* from Canada and Finland and those of *Neospora caninum*, *Hammondia heydorni*, *Toxoplasma gondii* and *H. hammondi* by using Clustal V (DNAStar, Madisin, Wisconsin, USA). Deletions and substitutions in the sequences relative to and within the clade of *B. acodoni*, *B. jellisoni*, *B. neotomofelis*, *B. darlingi* and *B. oryctofelisi* are indicated by black background. Sequences of the primer BdanjoRev and the Probe Bb11-12 are displayed in their complementary form. The probe Bb11-12 was established for a real-time PCR to detect *B. besnoiti*, but it is universal and can be used for the detection of all *Besnoitia* spp. mentioned here.

For the identification of suitable primer and probe regions, sequence data were downloaded from GenBank (sequence GenBank numbers are displayed in Fig. 1) and aligned using Clustal V (DNAStar, Madison, Wisconsin, USA).

## 3 Results

### 3.1 Specificity of the BdanjoRT1 real-time PCR

In-silico analysis revealed that probe Bb11-12, initially established to detect *B. besnoiti* DNA, was not only specific for *Besnoitia* spp. of ungulates, but also matched the ITS-1 sequences of further Besnoitia species. By contrast, primer sequences of BdanjoFwd and BdanjoRev (Fig. 1) were expected to match to sequences in the ITS-1 region of *B. akodoni, B. jellisoni, B. neotomofelis, B. darlingi* and *B. oryctofelisi*, but not to those of *Besnoitia* spp. of ungulates. In the cases of *B. oryctofelisi* and *B. darlingi*, there was only one miss-match (T instead of A) towards the 3′-end of the primer BdanjoRev sequence (Fig. 1).

**Fig. 2:**
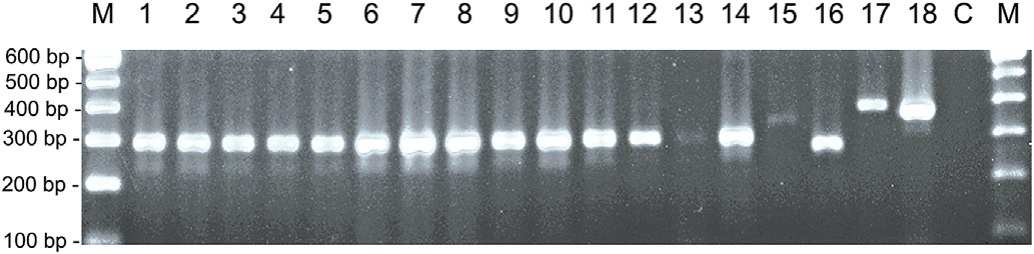
Coccidia-specific PCR to confirm the presence of DNA of various parasite species used to test the analytic specificity of the BdanjoRT1 real-time PCR. (1) *Besnoitia darlingi*, (2) *B. neotomofelis*, (3) *B. oryctofelisi*, (4) *B. besnoiti* (Evora isolate), (5) *B. bennetti* (Texas), (6) *B. tarandi* (Bt-CA-Quebec1), (7) *Toxoplasma gondii*, (8) *Hammondia hammondi*, (9) *Neospora caninum*, (10) *H. heydorni*, (11) *Cystoisospora felis*, (12) *C. rivolta*, (13) *C. burrowsi*, (14) *C. canis*, (15) *Sarcocystis cruzi* and (16) *Crytosporidium parvum*. Presence of (17) *Giardia duodenalis* and (18) *Tritrichomonas foetus* DNA was shown by amplification using species or genus-specific primers, respectively. C, negative control; M, marker.

The analytic specificity of the PCR assay was experimentally tested by using on the one hand DNA of *B. darlingi*, *B. neotomofelis* and *B. oryctofelisi* (i. e. DNA samples of parasites that were expected to be recognized by BdanjoRT1) and on the other hand DNA samples of *Besnoitia* spp. of ungulates (*B. besnoiti*, *B. tarandi*, *B. bennetti*) as well as DNA of related protozoan parasites *N. caninum*, *H. heydorni*, *T. gondii*, *H. hammondi*, *Cystoisospora* spp., *S. cruzi*, *C. parvum* and *G. duodenalis* and *T. foetus*. All these samples tested positive in an endpoint PCR targeting the SSU-rDNA of coccidian parasites (i.e. COC-1/COC-2 PCR positive; Fig. 2). In the BdanjoRT1 real-time PCR, only the DNA of *B. darlingi*, *B. neotomofelis* and *B. oryctofelisi* tested positive (Cq values of 19.7, 17.8, 20.6 respectively), while all remaining samples showed no specific amplification. All DNA samples, regardless of the test result in BdanjoRT1, showed no signs of inhibition as confirmed by the results of the IC2 PCR (range of Ct values: 31.7–33.2).

### 3.2 Analytic sensitivity and efficiency of the BdanjoRT1 real-time PCR

**Fig. 3:**
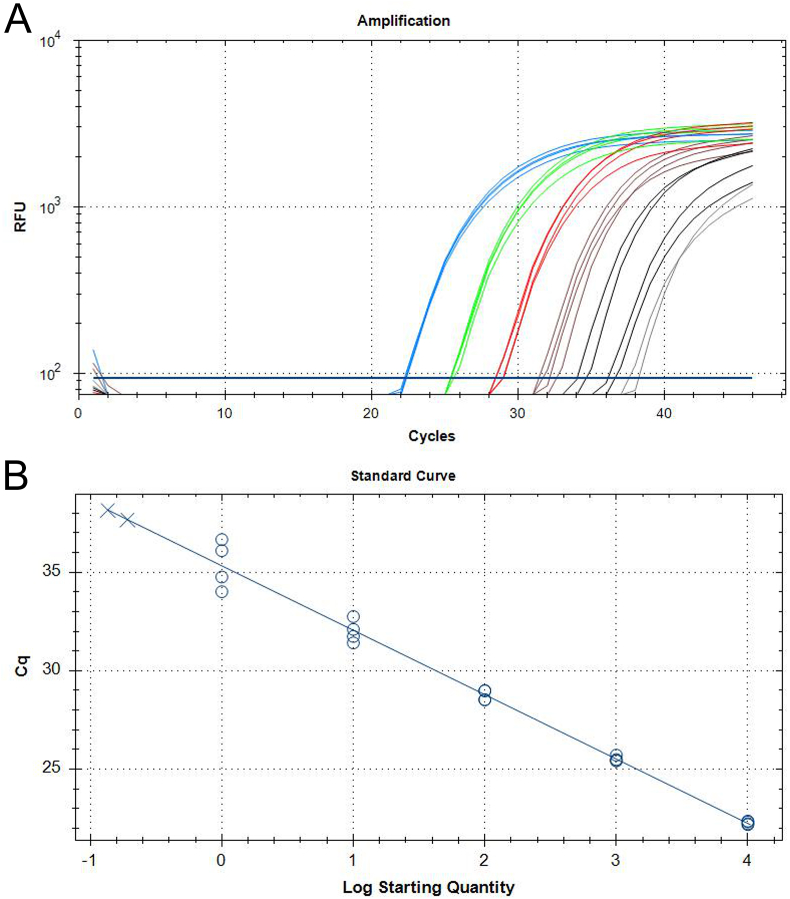
Analytic sensitivity (A) and standard curve (B) obtained for the threshold cycle (Cq) values obtained in the BdanjoRT1 real-time PCR using varying amounts of genomic *Besnoitia darlingi* DNA (approximately equivalent to the DNA content of 10.000 [blue], 1000 [green], 100 [red], 10 [brown] and 1 [black] *B. darlingi* tachyzoites) diluted in 100 ng/μl mouse DNA. Cq values used for regression are displayed as circles. Results on samples resembling DNA of 0.1 tachyzoite were not included in regression, since only two of four samples had reacted with a Cq value of 37.8 or 38.3 (displayed grey in A and as crosses in B). (For interpretation of the references to colour in this figure legend, the reader is referred to the Web version of this article.)

The analytic sensitivity of the BdanjoRT1 real-time PCR was assessed using *B. darlingi* tachyzoites isolated from infected CV-1 cell cultures. DNA isolated from a defined number of parasites was diluted in negative mouse DNA, resulting in concentrations sufficient to test DNA approximately equivalent to 10^4^ stages down to 0.1 stage per PCR reaction (two samples per tachyzoite concentration were analysed in duplicate). All samples approximately equivalent to the DNA content of 1 parasite per stage were detected with Cq values of 34.3–37.4 (Fig. 3A). Of the samples approximately equivalent to the DNA content of 0.1 tachyzoite, only two out of four samples reacted with a Cq value of 37.8 or 38.3. The efficiency of the BdanjoRT1 real-time PCR in this trial was 101.9%, the slope of the standard curve was −3.276 and the R^2^ value calculated for the standard curve was 0.99 (Fig. 3B). No PCR result showed a sign of inhibition as confirmed by the results obtained with the internal controls using the IC2 PCR (range of IC2 PCR Ct values: 32.4–33.4).

The analytic sensitivity was also assessed using faecal samples spiked with defined numbers of *B. darlingi* oocysts, approximately equivalent to concentrations of 100, 10, 1 or 0 oocysts per 150 mg of feline faeces (two faecal samples per concentration, analysed in duplicate). Since 150 mg of faeces resulted in a 100 μl DNA sample and 10 μl of this sample were analysed per PCR reaction, these concentrations resulted in DNA contents resembling 10, 1, 0.1 or 0 oocysts per PCR reaction. All samples with DNA equivalents of approximately 1 or 10 oocysts tested positive (1 oocyst: mean Cq value 34.3, range 34.1–35.1; range of IC2 PCR Cq: 31.7–32.8; 10 oocysts: mean Cq value 30.9, range 30.0–30.2; range of IC2 PCR Cq: 31.5–32.1). All samples with DNA equivalents of approximately 0.1 oocyst tested negative (Cq range of IC2 PCR: 31.6–32.6) and also all negative control fecal samples (Cq range of IC2 PCR: 32.2–32.5) were negative.

### 3.3 Real-time PCR results on GKO mouse tissues

Analysis of *B. darlingi* infected GKO mice used to establish a tachyzoite infected cell-culture showed a wide distribution of the stages in all examined organs (Table 2). The highest Cq values (Cq 34.1–34.2) were obtained in the brains of mice 5 or 8 days p.i., which suggests a low parasitic infestation. However, in an animal infected for 10 days, the Cq value was much lower (Cq 28.0), suggesting a higher concentration of parasite DNA resulting from ensuing multiplication. Lung was the organ in which the lowest Cq values (Cq 20.3–25.5) were observed in all animals, suggesting a very strong infestation of this organ in early infection.

**Table 2:** Real-time PCR results (Cq values) for tissue samples of ɣ-interferon-gene knockout mice inoculated with oocysts or an infectious homogenate of lung and heart tissue.

Organ	Animal V6/2 Oocysts infection, oral, 8 days p.i.	Animal V6/1 Oocysts infection, oral, 10 days p.i.	Animal V4/4 Homogenate of lung and heart of V6/2, intraperitoneal, 5 days p.i.	Mean
Brain	34.2	28.0	34.1	32.1
Heart	29.4	24.4	23.4	25.7
Lung	25.5	20.3	23.3	23.1
Liver	29.4	23.9	24.2	25.8
Kidney	31.8	25.8	25.1	27.6
Spleen	30.1	24.5	25.7	26.8
Skeletal muscle	31.8	22.4	27.1	27.1

## 4 Discussion

Besnoitiosis in rodents (*B. akodoni*, *B. jellisoni*, *B. neotomofelis*), marsupials (*B. darlingi*) and largomorphs (*B. oryctofelisi*) has been documented in North and South America (Dubey et al., 2003a,b; Dubey and Yabsley, 2010; Ernst et al., 1968; Venturini et al., 2002). *B. wallacei* was first described on Hawaii in its definitive host, the domestic cat, and experimental studies proposed rodents (mice, rats) as appropriate intermediate hosts (Frenkel, 1977). Further reports suggested the presence of similar *Besnoitia* spp. parasites in New Zealand, Australia, Japan and Kenya (Ito et al., 1978; Mason, 1980; McKenna and Charleston, 1980; Ng'ang'a et al., 1994). Thus, it is reasonable to wonder what other as-yet undescribed species may exist, and where they occur.

For some species domestic cats may serve as definitive hosts (*B. neotomofelis*, *B. darlingi*, *B. oryctofelisi*, *B. wallacei*), but this is not also true for other parasite species (*B. akodoni*, *B. jellisoni*) (Olias et al., 2011). In the cases of *B. neotomofelis*, *B. darlingi* and *B. oryctofelisi*, the intensity of oocyst shedding in domestic cats was low, suggesting that the domestic cat may not be the optimal definitive host for these parasites (Olias et al., 2011). In the case of *B. darlingi*, bobcats (*Lynx rufus*) were shown as natural definitive hosts (Verma et al., 2017). Thus, wild felids might serve as natural definitive hosts for other species of Besnoitia.

Strikingly, sequences of the ITS-1 rDNA region suggest that Besnoitia parasites in rodents (*B. akodoni, B. jellisoni, B. neotomofelis*), New World marsupials (*B. darlingi*) and largomorphs (*B. oryctofelisi*) are phylogenetically very closely related (Olias et al., 2011; Verma et al., 2017). In the present study, we made use of the limited variability of the ITS-1 region of these parasites and established a real-time PCR for the quantitative detection of DNA of these *Besnoitia* spp. Sequences deposited in GenBank™ suggested that this PCR should be able to detect DNA of *B. akodoni, B. neotomofelis*, *B. jellisoni*, *B. darlingi* and *B. oryctofelisi*. For *B. neotomofelis*, *B. darlingi*, and *B. oryctofelisi*, the expectations based on in-silico findings were confirmed by the positive reactions in the BdanjoRT1. For the remaining species, no DNA was available to study suitability of our PCR and confirmatory experiments can only be conducted after genetic material of these parasites becomes available.

The analytic sensitivity of the real-time PCR was similar to our previously published Besnoitia PCRs (Schares et al., 2011). A DNA equivalent of a single parasite was sufficient to yield a positive signal in the BdanjoRT1 real-time PCR. This was expected as in analogy to *T. gondii* (Guay et al., 1992), it can be assumed that there are multiple copies of the target DNA, the ITS-1 rDNA, in a single organism.

One possible application for this real-time PCR is the analysis of faeces of candidate definitive hosts. Here we describe a method purifying DNA directly from feline faeces capable of detecting DNA equivalent to what would be expected from 1 oocyst in 150 mg of faeces; a comparable sensitivity has been reported for a copro-PCR of 1–2 *T. gondii* oocysts per 200 mg of faeces (Salant et al., 2007, 2010). In a previous study, sucrose density centrifugation (flotation) and microscopical examination revealed a limit of detection of 250 oocysts per g of faeces (Rothe et al., 1985), i.e. 3-4 times less sensitive than the protocol reported here for *B. darlingi* oocysts. Unfortunately, we had a very limited number of oocysts and could do only a preliminary evaluation of this methodology. Further validation of this method on feline faeces is warranted, keeping in mind the effect of inhibitory substances; faeces types and concentrations of such inhibitors may markedly influence the outcome of DNA extraction and of the subsequent PCR. This was our experience, when we analysed fox faeces for *Echinococcus multilocularis* DNA (Maksimov et al., 2017). One advantage of the protocol established here is the inclusion of an internal control system to monitor potential inhibition in each sample tested.

The real-time PCR described here can also be used to examine tissues of intermediate hosts, as shown here with tissues of mice infected to generate a *B. darlingi* tachyzoite cell-culture isolate. Similar to experiences with other Besnoitia species, lung seems to be the predilection organ for multiplying tachyzoites, as shown by the notably low Cq values in real-time PCR (Gentile et al., 2012; Schares et al., 2019). Low Cq values in real-time PCR in the brain tissue were only observed in a mouse that had developed disease late, i.e. 10 days p.i. These findings are in accord with those reported previously on *B. darlingi* infected mice (Verma et al., 2017).

In summary, the BdanjoRT1 real-time PCR represents a sensitive tool useful for the testing of known and as yet unknown natural definitive and intermediate hosts of *B. akodoni*, *B. neotomofelis*, *B. jellisoni*, *B. darling, B. oryctofelisi* and related parasites. Owing to conservation of the particular ITS-1 region among related species of Besnoitia, these primers and probes may prove useful in detecting and characterizing as-yet undescribed species of Besnoitia in rodents, marsupials and lagomorphs.

## Funding

This research did not receive any specific grant from funding agencies in the public, commercial, or not-for-profit sectors.

## Declaration of competing interest

The study described is original and is not under consideration by any other journal. All authors approved the final manuscript and its submission. The authors declare that they have no conflict of interest.
